# A study on COVID-19 transmission dynamics: stability analysis of SEIR model with Hopf bifurcation for effect of time delay

**DOI:** 10.1186/s13662-020-02958-6

**Published:** 2020-09-24

**Authors:** M. Radha, S. Balamuralitharan

**Affiliations:** Department of Mathematics, College of Engineering and Technology, SRM Institute of Science and Technology, SRM Nagar, Kattankulathur, 603203 Kanchipuram, Chennai TN India

**Keywords:** 34D20, 34E05, 34K50, Covid19 Indian pandemic, SEIR, Stability, Hopf bifurcation, Sensitivity parameters

## Abstract

This paper deals with a general SEIR model for the coronavirus disease 2019 (COVID-19) with the effect of time delay proposed. We get the stability theorems for the disease-free equilibrium and provide adequate situations of the COVID-19 transmission dynamics equilibrium of present and absent cases. A Hopf bifurcation parameter *τ* concerns the effects of time delay and we demonstrate that the locally asymptotic stability holds for the present equilibrium. The reproduction number is brief in less than or greater than one, and it effectively is controlling the COVID-19 infection outbreak and subsequently reveals insight into understanding the patterns of the flare-up. We have included eight parameters and the least square method allows us to estimate the initial values for the Indian COVID-19 pandemic from real-life data. It is one of India’s current pandemic models that have been studied for the time being. This Covid19 SEIR model can apply with or without delay to all country’s current pandemic region, after estimating parameter values from their data. The sensitivity of seven parameters has also been explored. The paper also examines the impact of immune response time delay and the importance of determining essential parameters such as the transmission rate using sensitivity indices analysis. The numerical experiment is calculated to illustrate the theoretical results.

## Introduction

Globally COVID-19 coronavirus affects 235 countries and territories in which 31,561,520 peoples are affected and 970,688 died and recoverable populations 23,168,689. According to WHO (World Health Organization) data (on September 22, 2020), 5,562,663 have been affected and 88,935 people have died and 44,97,867 have been cured of diseases for the viruses in India. No mass transmission has taken place, and there is a possibility that it will peak in November and December 2020 and will start tapering slowly. Nevertheless, it will continue as an infectious virus, which will threaten the healthcare system by restoring in the future [1, 2]. The progressing COVID-19 episode, developed in Wuhan, China, has concerned more than 2600 lives starting on 24 February 2020 and represented a colossal danger to worldwide general wellbeing [3–8]. It has actually required different methods adding current situations and uncommon medical clinics and travel limitations to relieve the infection. Here we have indicated that coronavirus ailment has been engendered in the network of China quickly by revealed information and the Government of China make important strides, for example, occasion augmentation, travel limitation, hospitalization and isolation. These vital limitations have been useful to diminish the infection transmission among the populace and this is legitimized by information results [9–15]. It is earnest to give increasingly logical data to a superior comprehension of the novel coronavirus and promote control of the outbreak [16].

At the beginning of the time episode, it was dispersed, and connected to market places [17]. It has received outrageous measures to relieve flare-up. On 10 March 2020, the neighborhood legislature of Wuhan controlled every open traffic inside the city and shut all inbound and outbound transportation [18–24]. Muhammad Altaf Khan and Abdon Atangana discuss the model that the interaction between the bats and unidentified hosts are the reservoir of infections (seafood market) and the neighborhoods of people. The key cause of the infection is labeled seafood. The buying of goods from people’s seafood markets will infect asymptomatically or symptomatically. They reduced the model on the premise that the market in seafood has a sufficient source of infection to infect people [25]. Lim et al. discussed the spread of COVID-19 in South Korea through secondary transmission from the people who traveled from China [26]. Hu et al. revealed the potential for asymptomatic transmission by COVID-19 by examining the medical features of 24 asymptomatic patients developing near-contact infection [27]. The open frenzy progressing COVID-19 episode helps us in the records to remember the 1920 flu pandemic in London, United Kingdom. Besides, its attributes mellow side effects as a rule and short sequential interim are like pandemic flu, as opposed to the next two coronaviruses [28]. In 1918, critical extents of the passing were from pneumonia followed by flu contamination [29]. Hence, it may be sensible to return to the demonstrating system of the 1918 flu pandemic, and specifically, to catch the impacts of the individual response and government activity. We assume it will keep going for the following not many days for the occasion and shall refresh. The variables estimation might be evaluated after data have become accessible [30–33]. It contends all avoidance and limiting cases might be ordered up to three huge gatherings, that are portrayed as stage work and reaction work, separately. They likewise consider a COVID-19 transmission time of 14 days and gigantic resettlement from China [34]. A contact is an individual who encountered any of the accompanying exposures during the 2 days prior and the 14 days after the beginning of side effects of a plausible or affirmed case [35].

The median time before the onset of symptoms is 3 days, the shortest is 1 day, and the longest 24 days as recorded. These intervals have a significant role to play in understanding the dynamics of COVID-19 transmission. The mathematical model by Abdon Atangana agrees with its lock-down performance. The harmful effects of inadequate testing should be stated. The asymptomatic person tested may be good and spread the infection or may reach the virus within days after testing, and after the contradictory results the disease may spread further [36].

For COVID-19 among the human population and its stability we have proposed a SEIR pandemic model. Another scientific model in pestilence elements, known as the Warehouse theory, generally has been discussed for quite a while since it was proposed by Kermack and McKendrick in 1927. It incorporates a few essential improved models, for example, SIR, SIS, and SEIR, among which SEIR is an ordinary model that takes the incubation period into account. Giordano et al.’s findings show that the ongoing COVID-19 pandemic involves the combination of restrictive social-discriminatory behavior with widespread testing and contact monitoring. For the Italian COVID-19 epidemic, they estimate model parameters based on data dates from 20 February 2020 (day 1) to 5 April 2020 (day 46) and demonstrate the effect on the spread of the epidemic of progressive restrictions like the latest lock-down, slowly enforced as of 9 March 2020. They also model possible longer-term scenarios that show the impacts of various countermeasures, such as social separation and population-wide SARS-CoV-2 testing. The asymptomatic case is usually not reported to medical authorities as mentioned, and reported cases are typically only a fraction of the total number of symptomatic infectious people. The number of asymptomatic infectious cases and non-confirmed infectious cases and the number of recorded COVID cases in mainland China are discussed in this paper. The disparity between those diagnosed and those not diagnosed is significant. The former are being isolated usually and thus less likely is the infection to spread. This also helps clarify this descent. We mention the fatality rate and propagation of the disease misconception [37].

The SEIR, a widely utilized scourge model, can show the progressions of individuals between four states: Susceptible (S) (population not resistant to illness), Exposed (E) (population as of now in brooding), Infectious (I) (number of contamination effectively circling), and Recovered (R) (population not at this point irresistible because of confinement or in susceptibility or full recuperation). Here the population total size at time t is defined by N(t), with N(t) = S(t)+E(t)+I(t)+R(t). This system is portrayed by accompanying the nonlinear differential equations for the Indian current pandemic COVID19 [38]:

1 $$ \textstyle\begin{cases} \frac{{dS(t)}}{{dt}} = b + \gamma I(t) -(\mu + p)S(t)-\beta S(t)I(t), \\ \frac{{dE(t)}}{{dt}} = \beta S(t)I(t) - \mu E(t) - \eta E(t) R(t), \\ \frac{{dI(t)}}{{dt}}= (\eta + \sigma \beta ) E(t) - ( \alpha + \mu + \gamma ) I(t), \\ \frac{{dR(t)}}{{dt}}= p S(t) E(t) R(t) - ( \mu + \sigma \beta ) R(t). \end{cases} $$

The parameters p, b, *γ*, *β*, *μ*, *η*, *σ*, *α* are positive constants, p is the proportion of asymptomatic infection, b is the birth rate of people while newborn cells are created, *γ* is the incubation period of human infection, *β* is the transmission rate from one compartment to another compartment, *μ* is the death rate of people, *η* is the infectious period of symptomatic infection of people, *σ* is the infectious period of asymptomatic infection of people, *α* is the multiple of the transmissibility while infected cells are created from the viruses.

Transmission dynamics generating COVID-19 may require a duration of time delay *τ*, i.e. the delay of the immune system at time (days) *t* may be governed on the previous time $t-\tau $. We obtain an immune response of length of incubation period, $p S(t) E(t) R(t) = p S(t-\tau ) E(t-\tau ) R(t-\tau )$ and the duration of the patient being infectious. Tian-Mu Chen et al. [39] investigated the effect of including time delay to acquire the following nonlinear differential equations:

2 $$ \textstyle\begin{cases} \frac{{dS(t)}}{{dt}} = b + \gamma I(t) + \epsilon R(t) -(\mu + p)S(t)- \beta S(t)I(t), \\ \frac{{dE(t)}}{{dt}} = \beta S(t)I(t) - \mu E(t) - \eta E(t) R(t), \\ \frac{{dI(t)}}{{dt}}= (\eta + \sigma \beta ) E(t) - ( \alpha + \mu + \gamma ) I(t), \\ \frac{{dR(t)}}{{dt}}= p S(t-\tau ) E(t-\tau ) R(t-\tau ) - ( \epsilon + \mu + \sigma \beta ) R(t), \end{cases} $$

where *ϵ* is the latent period of human infection in population no longer infectious due to being fully recovered. The aim of the research work is to discuss the SEIR delay model in (2). If $\tau = 0$, Eq. (2) narrates the population inputs between size of population and number of initial infections. The COVID-19 basic reproduction number for the system (2) is defined by

$$ R_{0}= \frac{b \beta (\eta + \sigma \beta )}{\mu (\mu + p) (\alpha + \mu + \gamma )}. $$

We have likewise determined the basic reproduction number $R_{0}$ classical SIR model and we have seen that if $R_{0} < 1$ the disease does not proliferate into the population yet on the off chance that $R_{0} > 1$ infection will spread among the population. We presented an isolated SIR model and SEIR model portraying disease movement under the presumption that all contaminated individuals are separated after the hatching time frame so that they cannot taint others [40]. Ailment movement in these models is controlled by the basic reproduction number $R_{0}$, which is different from that for the standard SIR model. In the event that $R_{0} > 1$ (95%, ranges 1.4 to 3.9), at that point the quantity of inertness contaminated people exponentially develops. Be that as it may, if $R_{0} < 1$, at that point the number of contaminated behaves exponentially. This investigation of $R_{0}$ catches the course of COVID-19 flare-up and subsequently reveals insight and understanding of the patterns of the flare-up and gives some preventive, measure not to spread the COVID-19 malady (97%, ranges 2.47 to 3.9). This portrays the normal number of recently contaminated cells produced from one tainted cell toward the start of the irresistible procedure. The current scenario will evolve to account for these continuing advancements as new drugs and vaccines are being developed and tested [41].

## Preliminaries

Let ${S(t)=C ( [-\tau,0 ];\mathbb{R} )}$ be the continuous norm function of Banach space mappings. The initial conditions for the model (2) are given as follows:

3 $$ \textstyle\begin{cases} S (t )\geq 0,\qquad E (t )\geq 0, \qquad I (t ) \geq 0, \qquad R (t )\geq 0, \quad t\in [-\tau,0 ], \\ S (0 )>0, \qquad E (0 )>0, \qquad I (0 )>0, \qquad R (0 )>0. \end{cases} $$

Let $(S(t), E(t), R(t))$ be three main variables of the system with initial conditions and verify that there is a unique solution. The accompanying lemma is helpful for examining the positivity of the bounded solutions.

### Lemma 2.1

*In the system* $(S(t), E(t), R(t))$*of* (2) *with initial conditions* (3), *we state that*

$$ \lim \sup S (t )_{{t\rightarrow +\infty }}\leq \frac{b}{{\mu + p}}. $$

### Proof

If there is $t_{1}>0$ with the end goal that $S(t_{1})> \frac{b}{{\mu + p}}$ and $\overset{\hbox{\tiny$\bullet$}}{S}(t_{1})>0$, then we have

$$ \overset{\hbox{\tiny$\bullet$}}{S}(t_{1})=b -(\mu + p)S(t)-\beta S(t)I(t)\leq -\beta S(t)I(t) \leq 0. $$

Hence we have utilized $S(t_{1})> \frac{{b}}{{\mu + p}}$. This is an inconsistency to $\overset{\hbox{\tiny$\bullet$}}{S}(t_{1})>0$. Along these lines, Lemma 2.1 is verified. □

### Lemma 2.2

*Let* $(S(t), E(t),I(t),R(t))$*be the system* (2) *with initial conditions* (3). *At that point* $(S(t), E(t), I(t))$*and* $R(t)$*are certain and there exists a positive constant* $\Gamma >0$, *to such an extent that* $S(t)<\Gamma,E(t)<\Gamma,I(t)<\Gamma $*and* $R(t)<\Gamma $*at an adequately huge time t*.

### Proof

Considering Eq. (2), we get

$$\begin{aligned} &S (t )=S (0 )e^{-\int ^{t}_{0} ((\mu + p)- \beta I(\epsilon ) )\,d\epsilon } + \int ^{t}_{0} b e^{-\int ^{t}_{ \eta } ((\mu + p)-\beta I(\epsilon ) ) \,d \epsilon } \,d\eta, \\ &E (t )=E (0 )e^{-\int ^{t}_{0} (\mu + \eta R (\epsilon ) ) \,d\epsilon }+ \int ^{t}_{0} \beta S (\eta )I (\eta )e^{-\int ^{t}_{0} (\mu + \eta R (\epsilon ) ) \,d\epsilon } \,d\eta, \\ &I (t )=I (0 )e^{-(\mu +\alpha +\gamma )t}+ \int ^{t}_{0} (\eta +\beta \sigma )E (\eta )e^{-( \mu +\alpha +\gamma ) (t-\eta )} \,d\eta, \\ &R (t )=R (0 )e^{- (\sigma \beta + \epsilon +\mu )t}+ \int ^{t}_{0}pS (\eta -\tau )E (\eta -\tau )R (\eta -\tau )e^{ ( \sigma \beta +\epsilon +\mu )t}\,d\eta. \end{aligned}$$

It is anything but difficult to see that ${S(t)}$ is positive on the existence interval. At that point, we demonstrate that ${E(t)}$ is positive. Truth be told, let ${t_{1}}>0$ be the first run through to such an extent that ${E(t_{1})=0}$. From Eq. (2), we get

$$ {I(t)=I(0)e^{-(\mu +\alpha +\gamma )t_{1}}+ \int ^{t_{1}}_{0} ( \eta + \sigma \beta )E(\eta )e^{-(\alpha + \mu + \gamma )(t- \eta )}\,d\eta >0}. $$

Then again, from the second equation of (2), we have $\overset{\hbox{\tiny$\bullet$}}{E}(t_{1})=\beta S(t_{1})I(t_{1})>0$. This implies ${E(t)<0}$ for $t\in (t_{1}-\xi,t_{1})$, where *ξ* is a subjectively small positive constant, which prompts an inconsistency. It is follows that $E(t)>0$ and $I(t)>0$. By the comparative contention as mentioned above, it is difficult to see that $R(t)$ is positive. Here, we discuss the contentions for an extreme solution of (2).

Here ${N(t) = S(t)+E(t) + (\frac{{\mu }}{{2 (\eta + \sigma \beta )}})I(t)+ (\frac{{\eta (\mu + p)}}{{pb}})R(t+\tau ) }$, and we assume $q=\min \{ (\mu + p),\frac{\mu }{2},(\alpha + \mu + \gamma ), ( \epsilon +\mu + \sigma \beta ) \} $.

From (2), we get

$$\begin{aligned} \frac{{d}}{{dt}} \bigl[N (t ) \bigr]={}&b -(\mu + p)S(t)- \biggl(\frac{\mu }{2}\biggr)E(t)- \eta E(t)R(t)- \biggl(\frac{\mu (\alpha + \mu + \gamma )}{2 (\eta + \sigma \beta )}\biggr)I(t) \\ &{}+\biggl(\frac{\eta (\mu + p)}{b}\biggr)S(t)E(t)R(t)- \biggl(\frac{\eta (\epsilon +\mu + \sigma \beta ) (\mu + p)}{pb}\biggr)R(t+ \tau ) \\ \leq{}& b-(\mu + p)S(t)-\biggl(\frac{\mu }{2}\biggr)E(t)- \biggl(\frac{\mu (\alpha + \mu + \gamma )}{2 (\eta + \sigma \beta )}\biggr)I(t) \\ &{}- \biggl(\frac{\eta (\epsilon +\mu + \sigma \beta ) (\mu + p)}{pb}\biggr)R(t+ \tau ) \\ < {}&b -q \biggl[S(t)+E(t) + \biggl(\frac{{\mu }}{{2 (\eta + \sigma \beta )}}\biggr)I(t)+\biggl( \frac{{\eta (\mu + p)}}{{p b}}\biggr)R(t+\tau ) \biggr] \\ ={}&b-qN. \end{aligned}$$

Along these lines, $N(t)<\frac{b}{(\mu + p)\eta }$ for all large *t*. Subsequently, $S(t),E(t),I(t)$ and $R(t)$ are at last limited by any positive constant Γ. Hence, we finish the verification of Lemma 2.2. □

## Theorems for stability analysis

There are three equilibria for system (2):

(i) *COVID-19 infection free equilibrium:* $E_{0}= (\frac{b}{(\mu + p)},0,0,0 )$.
(ii) *COVID-19 infection absent equilibrium:* $E_{1}= (\frac{\mu (\alpha + \mu + \gamma )}{\beta } (\eta + \sigma \beta ), \frac{b \beta (\eta + \sigma \beta )-\mu (\mu + p) (\alpha + \mu + \gamma )}{\mu \beta (\eta + \sigma \beta )}, \frac{ b\beta (\eta + \sigma \beta )- \mu (\mu + p) (\alpha + \mu + \gamma )}{\mu \beta (\alpha + \mu + \gamma )},0 )$.
(iii) *COVID-19 infection present equilibrium:* $\bar{E}= (\bar{E_{1}}, \bar{E_{2}}, \bar{E_{3}}, \bar{E_{4}} )$,
  where
  $\bar{E_{1}}= \frac{p (\alpha + \mu + \gamma )b-\beta (\eta + \sigma \beta ) (\epsilon +\mu + \sigma \beta )}{p (\mu + p) (\alpha + \mu + \gamma )}$, $\bar{E_{2}}= \frac{ (\epsilon +\mu + \sigma \beta ) (\mu + p) (\alpha + \mu + \gamma )}{p (\alpha + \mu + \gamma ) b-\beta (\eta + \sigma \beta ) (\epsilon +\mu +\sigma \beta )}$, $\bar{E_{3}}= \frac{ (\eta + \sigma \beta ) (\epsilon +\mu + \sigma \beta ) (\mu + p)}{p (\alpha + \mu + \gamma ) b-\beta (\eta + \sigma \beta ) (\epsilon +\mu + \sigma \beta )}$, $\bar{E_{4}}=\frac{1}{\eta } [ \frac{\beta (\eta + \sigma \beta ) (p (\alpha + \mu + \gamma ) b-\beta (\eta + \sigma \beta ) (\epsilon +\mu + \sigma \beta ) )}{p (\mu + p)(\alpha + \mu + \gamma )^{2}}- \mu ]$.

### Stability of COVID-19 infection free equilibrium

The nonlinear differential equation of (2) at the point $E_{0}$ is

4 $$ \textstyle\begin{cases} \frac{{dS(t)}}{{dt}}=-(\mu + p)S(t)-\frac{\beta b}{(\mu + p)}I(t), \\ \frac{{dE(t)}}{{dt}}=-\mu E(t)+\frac{\beta b}{(\mu + p)}I(t), \\ \frac{{dI(t)}}{{dt}}= (\eta + \sigma \beta )E(t)-( \alpha + \mu + \gamma )I(t), \\ \frac{{dR(t)}}{{dt}}=- (\epsilon +\mu + \sigma \beta )R(t). \end{cases} $$

The polynomial equation for (4) is

5 $$\begin{aligned} & \bigl[\lambda + (\epsilon +\mu + \sigma \beta ) \bigr] \bigl[ \lambda +(\mu + p)\bigr] \\ &\quad{} \biggl[\lambda ^{2}+\bigl(\mu +(\alpha + \mu + \gamma )\bigr) \lambda + \mu (\alpha + \mu + \gamma )- \frac{ (\eta + \sigma \beta ) \beta b}{(\mu + p)} \biggr]=0. \end{aligned}$$

Two of the roots of the polynomial equation (5) are $\lambda _{1}=- (\epsilon +\mu + \sigma \beta )$, $\lambda _{2}=-(\mu + p)$. The other roots are calculated by

6 $$ \lambda ^{2}+\bigl(\mu +(\alpha + \mu + \gamma )\bigr) \lambda +\mu (\alpha + \mu + \gamma )- \frac{ (\eta + \sigma \beta )\beta b}{(\mu + p)}=0. $$

If $R_{0}<1$, then $\mu (\alpha + \mu + \gamma )- \frac{ (\eta + \sigma \beta )\beta b}{(\mu + p)}>0$, and $(\mu +(\alpha + \mu + \gamma ))^{2}-4 (\mu (\alpha + \mu + \gamma )-\frac{ (\eta + \sigma \beta )\beta b}{(\mu + p)} )>0$. We have

$$ \lambda _{3,4}= \frac{-(\mu +(\alpha + \mu + \gamma ))\pm \sqrt{(\mu +(\alpha + \mu + \gamma ))^{2}-4 (\mu (\alpha + \mu + \gamma )-\frac{ (\eta + \sigma \beta )\beta b}{(\mu + p)} )}}{2}. $$

Equation (6) has negative real roots. It obeys the accompanying theorem. If $R_{0}<1$, then $E_{0}$ is seen to be locally asymptotic stable by developing a Lyapunov functional. If $R_{0}>1$, then $E_{0}$ is unstable.

#### Theorem 3.1

*If* $R_{0}<1$, *then prove that* $E_{0}$*is globally asymptotic stable*.

#### Proof

For the Lyapunov functional

$$\begin{aligned} V={}&\frac{1}{2} \biggl[S(t)- \frac{ (\epsilon +\mu + \sigma \beta )}{(\mu + \eta )} \biggr]^{2}+ \frac{ (\epsilon +\mu + \sigma \beta )}{(\mu + \eta )}E(t)+mI(t)+\frac{\eta }{p}R(t)\\ &{}+\eta \int ^{t}_{t-\tau }S(\theta )E( \theta )R(\theta )\,d \theta, \end{aligned}$$

where $m >0$, we have

$$\begin{aligned} V'|_{(5)}={}& \biggl[S(t)-\frac{b}{(\mu + p)} \biggr] \biggl[-(\mu + p) \biggl(S(t)-\frac{b}{(\mu + p)} \biggr)-\beta S(t)I(t) \biggr] \\ &{}+\frac{b}{(\mu + p)} \bigl[\beta S(t)I(t)-\mu E(t)-\eta E(t)R(t) \bigr] \\ &{}+m \bigl[ (\eta + \sigma \beta ) E(t)-(\alpha + \mu + \gamma ) I(t) \bigr]- \biggl(\frac{\eta (\epsilon +\mu + \sigma \beta )}{p}\biggr)R(t)+\eta S(t)E(t)R(t). \end{aligned}$$

Since ${\beta S(t)I(t)=\beta I(t) [S(t)-\frac{b}{(\mu + p)} ]+ \frac{\beta b}{(\mu + p)}I(t)}$, we have

$$\begin{aligned} V'|_{(5)}={}&{-}(\mu + p) \biggl[S(t)-\frac{b}{(\mu + p)} \biggr]^{2}- \beta I(t) \biggl[S(t)-\frac{b}{(\mu + p)} \biggr]^{2}\\ &{}+\eta S(t)R(t) \biggl[S(t)-\frac{b}{(\mu + p)} \biggr] \\ &{}- \biggl[\frac{b \mu }{(\mu + p)}- (\eta + \sigma \beta ) m \biggr]E(t)- \biggl[( \alpha + \mu + \gamma ) m- \frac{\beta b^{2}}{(\mu + p)^{2}} \biggr]I(t)\\ &{}- \biggl[\frac{\eta (\epsilon +\mu + \sigma \beta )}{p}\biggr]R(t). \end{aligned}$$

Hence $R_{0}<1$ decreases to $\frac{b \mu }{ (\eta + \sigma \beta ) (\mu + p)}-\frac{\beta b^{2}}{(\alpha + \mu + \gamma ) (\mu + p)^{2}}>0$, $(m\in [ \frac{\beta b^{2}}{(\alpha + \mu + \gamma ) (\mu + p)^{2}}, \frac{b \mu }{ (\eta + \sigma \beta ) (\mu + p)} ] )$, such that $\frac{b \mu }{(\mu + p)}- (\eta + \sigma \beta ) m>0$ and $(\alpha + \mu + \gamma ) m-\frac{\beta b^{2}}{(\mu + p)^{2}}>0$.

Letting $S(t),E(t),R(t)$ be positive and $S(t)\leq \frac{b}{(\mu + p)}$ holds, we have $V'|_{(5)}\leq 0$, and $V'|_{(5)}=0$ iff $(S(t),E(t),I(t),R(t))= (\frac{b}{(\mu + p)},0,0,0 )$. □

### Stability of COVID-19 infection absent equilibrium

Letting $E_{1}= (\tilde{S},\tilde{E},\tilde{I},0 )= ( \frac{\mu (\alpha + \mu + \gamma )}{\beta (\eta + \sigma \beta )}, \frac{b \beta -\mu (\mu + p) (\alpha + \mu + \gamma )}{\mu \beta (\alpha + \mu + \gamma )}, \frac{b \beta (\eta + \sigma \beta )}{\mu \beta (\alpha + \mu + \gamma )},0 )$, the linearized form of equations of system (2) at ${E_{1}}$ is

7 $$ \textstyle\begin{cases} \frac{{dS(t)}}{{dt}}=- ((\mu + p)+\beta \tilde{I} )S(t)- \beta \tilde{S}I(t), \\ \frac{{dE(t)}}{{dt}}=\beta \tilde{I}S(t)-\mu E(t)+\beta \tilde{S}I(t)- \eta \tilde{E}R(t), \\ \frac{{dI(t)}}{{dt}}= (\eta + \sigma \beta ) E(t)-( \alpha + \mu + \gamma ) I(t), \\ \frac{{dR(t)}}{{dt}}=p\tilde{S}\tilde{E}R(t-\tau )- (\epsilon + \mu + \sigma \beta ) R(t). \end{cases} $$

The characteristic polynomial equation of (7) is

$$\bigl(\lambda -p \tilde{S}\tilde{E}e^{-\lambda \tau }+ ( \epsilon +\mu + \sigma \beta ) \bigr) \bigl(\lambda ^{3}+a_{1} \lambda ^{2}+a_{2}\lambda +a_{3} \bigr)=0, $$

where

$$\begin{aligned}& a_{1}=\mu +(\alpha + \mu + \gamma )+(\mu + p)+ \frac{b \beta (\eta + \sigma \beta )-\mu (\mu + p) (\alpha + \mu + \gamma )}{\mu (\alpha + \mu + \gamma )}, \\& a_{2}=\bigl(\mu +(\alpha + \mu + \gamma )\bigr) \biggl((\mu + p)+ \frac{b \beta (\eta + \sigma \beta )-\mu (\mu + p) (\alpha + \mu + \gamma )}{\mu (\alpha + \mu + \gamma )} \biggr), \\& a_{3}= b \beta (\eta + \sigma \beta )-\mu (\mu + p) ( \alpha + \mu + \gamma ). \end{aligned}$$

First we obtain

8 $$\begin{aligned} \lambda ^{3}+a_{1}\lambda ^{2}+a_{2}\lambda +a_{3}=0. \end{aligned}$$

Clearly, if $R_{0}>1$, we have $a_{1}=\mu +(\alpha + \mu + \gamma )+ (\mu + p)+ \frac{ b \beta (\eta + \sigma \beta )-\mu (\mu + p) (\alpha + \mu + \gamma )}{\mu (\alpha + \mu + \gamma )}>0$ and

$$\begin{aligned} &\phantom{a_{1}a_{2}-{}}a_{3}=b \beta (\eta + \sigma \beta )-\mu (\mu + p) ( \alpha + \mu + \gamma ), \\ &a_{1}a_{2}-a_{3}= \biggl(\mu +(\alpha + \mu + \gamma )+(\mu + p)+ \frac{b \beta (\eta + \sigma \beta )-\mu (\mu + p) (\alpha + \mu + \gamma )}{\mu (\alpha + \mu + \gamma )} \biggr) \\ &\phantom{a_{1}a_{2}-a_{3}=}{}\times\bigl(\mu +(\alpha + \mu + \gamma )\bigr) \\ &\phantom{a_{1}a_{2}-a_{3}=}{}\times \biggl((\mu + p)+ \frac{b \beta (\eta + \sigma \beta )-\mu (\mu + p) (\alpha + \mu + \gamma )}{\mu (\alpha + \mu + \gamma )} \biggr) \\ &\phantom{a_{1}a_{2}-a_{3}=}{}-\bigl(b \beta (\eta + \sigma \beta )-\mu (\mu + p) ( \alpha + \mu + \gamma ) \bigr) \\ &\phantom{a_{1}a_{2}-a_{3}}=\mu ^{2} (\mu + p)+ \frac{\mu (b \beta (\eta + \sigma \beta )- \mu (\mu + p) (\alpha + \mu + \gamma ))}{(\alpha + \mu + \gamma )}+ \mu (\mu + p) (\alpha + \mu + \gamma ) \\ &\phantom{a_{1}a_{2}-a_{3}=}{}+ \biggl((\alpha + \mu + \gamma )+(\mu + p)+ \frac{b \beta (\eta + \sigma \beta )-\mu (\mu + p) (\alpha + \mu + \gamma )}{\mu (\alpha + \mu + \gamma )} \biggr) \\ &\phantom{a_{1}a_{2}-a_{3}=}{}\times\biggl(\mu (\mu + p)+ \frac{b \beta (\eta + \sigma \beta )-\mu (\mu + p) (\alpha + \mu + \gamma )}{\mu (\alpha + \mu + \gamma )} \\ &\phantom{a_{1}a_{2}-a_{3}=}{}+(\alpha + \mu + \gamma ) (\mu + p)+ \frac{b \beta (\eta + \sigma \beta )-\mu (\mu + p) (\alpha + \mu + \gamma )}{\mu (\alpha + \mu + \gamma )} \biggr)>0. \end{aligned}$$

By the Routh–Hurwitz criteria, (8) has no positive roots. So, we investigate the other polynomial equation

9 $$ \lambda -p\tilde{S}\tilde{E}e^{-\lambda \tau }+ ( \epsilon +\mu + \sigma \beta )=0. $$

For $\tau =0$, $\lambda = \frac{\beta (\eta + \sigma \beta ) p (\alpha + \mu + \gamma ) b-\beta ^{2} (\eta + \sigma \beta )^{2} (\epsilon +\mu + \sigma \beta )-\mu p (\mu + p) (\alpha + \mu + \gamma )^{2}}{\beta ^{2} (\eta + \sigma \beta )^{2}}$. Obviously, if $R_{0}<1+ \frac{\beta ^{2} (\eta + \sigma \beta )^{2} (\epsilon +\mu + \sigma \beta )}{\mu p (\mu + p) (\alpha + \mu + \gamma )^{2}}$, then $\phi <0$, which illustrates the roots of (9) for some $\phi >0$ and $\tau >0$. From (9), we have

10 $$ \textstyle\begin{cases} \hphantom{+\mu + \sigma \beta ))\,\,} \phi =- \frac{(\alpha + \mu + \gamma ) p (b \beta (\eta + \sigma \beta )-\mu (\mu + p) (\alpha + \mu + \gamma ))}{\beta ^{2} (\eta + \sigma \beta )^{2}}\sin \phi \tau, \\ (\epsilon +\mu + \sigma \beta )= \frac{(\alpha + \mu + \gamma ) p(s\beta (\eta + \sigma \beta )-\mu (\mu + p) (\alpha + \mu + \gamma ) )}{\beta ^{2}k^{2}}\cos \phi \tau, \end{cases} $$

which implies that $\phi ^{2}=p^{2} [ \frac{(\alpha + \mu + \gamma ) p(b\beta (\eta + \sigma \beta )-\mu (\mu + p) (\alpha + \mu + \gamma ))}{\beta ^{2} (\eta + \sigma \beta )^{2}} ]^{2}- ( \epsilon +\mu + \sigma \beta )^{2}$.

Note that if $1< R_{0}<1+ \frac{\beta ^{2} (\eta + \sigma \beta )^{2} (\epsilon +\mu + \sigma \beta )}{\mu p (\mu + p) (\alpha + \mu + \gamma )^{2}}$, then $\phi ^{2}<0$. If $1< R_{0}<1+ \frac{\beta ^{2} (\eta + \sigma \beta )^{2} (\epsilon +\mu + \sigma \beta )}{\mu p (\mu + p) (\alpha + \mu + \gamma )^{2}}$, then the COVID-19 infection $E_{1}$ is locally asymptotic stable. If $1< R_{0}>1+ \frac{\beta ^{2} (\eta + \sigma \beta )^{2} (\epsilon +\mu + \sigma \beta )}{\mu p (\mu + p) (\alpha + \mu + \gamma )^{2}}$, then the COVID-19 infection $E_{1}$ is unstable.

### Stability of COVID-19 infection present equilibrium

In COVID-19 infection, the effects of time delay *τ* is a bifurcation parameter and it goes through a stationary values. The COVID-19-present equilibrium occurs direct stability and Hopf bifurcation. As a matter of first importance, we interpret the equilibrium $\bar{E}=(\bar{S},\bar{E},\bar{I},\bar{R})$ of system (2) to the source. Let $S_{1}(t)=S(t)- \bar{S},E_{1}(t)=E(t)- \bar{E},I_{1}(t)=I(t)- \bar{I},R_{1}(t)=R(t)- \bar{R}$. For effortlessness, we likewise use $S(t),E(t),I(t),R(t)$ rather than $S_{1}(t),E_{1}(t),I_{1}(t),R_{1}(t)$. The system (2) becomes

11 $$ \textstyle\begin{cases} \frac{{dS(t)}}{{dt}}=- ((\mu + p)+\beta \bar{I} )S(t)- \beta S(t)I(t)-\beta \bar{S}I(t), \\ \frac{{dE(t)}}{{dt}}=\beta S(t)I(t)+\beta \bar{I}S(t)-(\mu +\eta \bar{R})E(t)+\beta \bar{S}I(t)-\eta \bar{E}R(t)-\eta E(t)R(t), \\ \frac{{dI(t)}}{{dt}}= (\eta + \sigma \beta ) E(t)-( \alpha + \mu + \gamma ) I(t), \\ \frac{{dR(t)}}{{dt}}=p S(t-\tau )E(t-\tau )R(t-\tau )+p\bar{E}S(t- \tau )R(t-\tau )+p\bar{S}E(t-\tau )R(t-\tau ) \\ \phantom{\frac{{dR(t)}}{{dt}}=}{} +p\bar{S}\bar{E}R(t-\tau ) +p\bar{R}S(t-\tau )E(t-\tau )+p \bar{E}\bar{R}S(t-\tau )+p\bar{S}\bar{R}E(t-\tau ) \\ \phantom{\frac{{dR(t)}}{{dt}}=} {} - (\epsilon +\mu + \sigma \beta ) R(t). \end{cases} $$

Then the origin $(0,0,0,0)^{T}$ is a steady state of (11) and the linearized system of equation (11) at the origin is

12 $$ \textstyle\begin{cases} \frac{{dS(t)}}{{dt}}=- ((\mu + p)+\beta \bar{I} )S(t)- \beta S(t)I(t)-\beta \bar{S}I(t), \\ \frac{{dE(t)}}{{dt}}=\beta S(t)I(t)+\beta \bar{I}S(t)-(\mu + \eta \bar{R})E(t)+\beta \bar{S}I(t)-\eta \bar{E}R(t)-\eta E(t)R(t), \\ \frac{{dI(t)}}{{dt}}= (\eta + \sigma \beta ) E(t)-( \alpha + \mu + \gamma ) I(t), \\ \frac{{dR(t)}}{{dt}}=pS(t-\tau )E(t-\tau )R(t-\tau )+p\bar{E}S(t- \tau )R(t-\tau )+p\bar{S}E(t-\tau )R(t-\tau ) \\ \phantom{\frac{{dR(t)}}{{dt}}=}{} +p\bar{S}\bar{E}R(t-\tau ) +p\bar{R}S(t-\tau )E(t-\tau )+p \bar{E}\bar{R}S(t-\tau )+p\bar{S}\bar{R}E(t-\tau ) \\ \phantom{\frac{{dR(t)}}{{dt}}=}{} - (\epsilon +\mu + \sigma \beta ) R(t). \end{cases} $$

The trivial solution of Eq. (12) is asymptotic stable and Eq. (11) is locally asymptotic stable. The strength of the polynomial equation (12) is given by

13 $$\begin{aligned} \Omega (\lambda )=\lambda ^{4}+x_{1} \lambda ^{3}+x_{2}\lambda ^{2}+x_{3} \lambda +x_{4}-\bigl(x_{5}\lambda ^{3}+x_{6} \lambda ^{2}+x_{7}\lambda +x_{8} \bigr)e^{- \lambda \tau }, \end{aligned}$$

where

$$\begin{aligned}& x_{1}= (\epsilon +\mu + \sigma \beta )+(\mu + p)+\beta \bar{I}+(\alpha + \mu + \gamma )+\mu +\eta \bar{R}, \\& \begin{aligned} x_{2}&= (\epsilon +\mu + \sigma \beta ) (\mu + p)+ ( \epsilon +\mu + \sigma \beta )\beta \bar{I}+ (\epsilon + \mu + \sigma \beta ) (\alpha + \mu + \gamma ) \\ &\quad {}+(\mu + p) ( \alpha + \mu + \gamma )+\beta \bar{I} (\alpha + \mu + \gamma )+(\mu + \eta \bar{R})\bigl( (\epsilon +\mu + \sigma \beta ) +(\mu + p)+ \beta \bar{I}\bigr), \end{aligned} \\& \begin{aligned} x_{3}&= (\epsilon +\mu + \sigma \beta ) (\mu + p) ( \alpha + \mu + \gamma )+ (\epsilon +\mu + \sigma \beta ) \beta \bar{I} (\alpha + \mu + \gamma ) \\ &\quad {}+(\mu +\eta \bar{R})\bigl( ( \epsilon +\mu + \sigma \beta ) (\mu + p)+ (\epsilon +\mu + \sigma \beta )\beta \bar{I}\bigr)+ (\eta + \sigma \beta )\beta ^{2}\bar{S}\bar{I}, \end{aligned} \\& x_{4}= (\epsilon +\mu + \sigma \beta ) (\eta + \sigma \beta )\beta ^{2}\bar{S}\bar{I}, \\& x_{5}= (\epsilon +\mu + \sigma \beta ), \\& x_{6}= (\epsilon +\mu + \sigma \beta ) (\mu + p)+ ( \epsilon +\mu + \sigma \beta )\beta \bar{I}+ (\epsilon + \mu + \sigma \beta ) (\alpha + \mu + \gamma )+\mu ( \epsilon +\mu + \sigma \beta ), \\& \begin{aligned} x_{7}&= (\epsilon +\mu + \sigma \beta ) (\mu + p) ( \alpha + \mu + \gamma )+ (\epsilon +\mu + \sigma \beta ) \beta \bar{I}(\alpha + \mu + \gamma ) \\ &\quad {}+(\mu +\eta \bar{R})\bigl( ( \epsilon +\mu + \sigma \beta ) (\mu + p)+ (\epsilon +\mu + \sigma \beta )\beta \bar{I}\bigr) \\ &\quad {}- (\epsilon +\mu + \sigma \beta ) \eta \bar{R}\bigl((\mu + p)+\beta \bar{I}+(\alpha + \mu + \gamma )\bigr), \end{aligned} \\& x_{8}= (\epsilon +\mu + \sigma \beta ) (\eta + \sigma \beta )\beta ^{2}\bar{S}\bar{I}- (\epsilon +\mu + \sigma \beta ) (\mu + p) (\alpha + \mu + \gamma ) \eta \bar{R}. \end{aligned}$$

#### Theorem 3.2

*If the solution of* (12) *is locally asymptotic stable*, *then* $\tau =0$*and* $R_{0}>1+ \frac{\beta ^{2} (\eta + \sigma \beta )^{2} (\epsilon +\mu + \sigma \beta )}{\mu p (\mu + p) (\alpha + \mu + \gamma )^{2}}$.

#### Proof

Let $\tau =0$. From (13),

14 $$\begin{aligned} \lambda ^{4}+(x_{1}+x_{5}) \lambda ^{3}+(x_{2}-x_{6})\lambda ^{2}+(x_{3}-x_{7}) \lambda +x_{4}-x_{8}=0. \end{aligned}$$

Since $R_{0}>1+ \frac{\beta ^{2} (\eta + \sigma \beta )^{2} (\epsilon +\mu + \sigma \beta )}{\mu p (\mu + p) (\alpha + \mu + \gamma )^{2}}$, $\bar{S}>0$, $\bar{E}>0$, $\bar{I}>0$, $\bar{R}>0$.

By the method of the Routh–Hurwitz criteria, we get

$$\begin{array}{c} x_{9}=x_{1}-x_{5}=\beta \bar{I}+\eta \bar{R}+3\mu +p+\alpha +\gamma >0,\\ \begin{array}{rl} x_{10} & =(x_{1}-x_{5})(x_{2}-x_{6})-(x_{3}-x_{7})\\ & =((\mu +p)+\beta \bar{I}+(\alpha +\mu +\gamma )+\mu +\eta \bar{R}[(\mu +p)(\alpha +\mu +\gamma )+\beta \bar{I}(\alpha +\mu +\gamma )\\ & \;+(\mu +\eta \bar{R})\left((\epsilon +\mu +\sigma \beta )+(\mu +p)+\beta \bar{I}\right)-\mu (\epsilon +\mu +\sigma \beta )]\\ & \;-(\epsilon +\mu +\sigma \beta )(\eta +\sigma \beta )\beta^{2}\bar{S}\bar{I}-(\epsilon +\mu +\sigma \beta )\eta \bar{R}\left((\mu +p)+\beta \bar{I}+(\alpha +\mu +\gamma )\right)\\ & \;\left((\mu +p)+\beta \bar{I}+(\alpha +\mu +\gamma )\right)[(\mu +p)(\alpha +\mu +\gamma )+(\alpha +\mu +\gamma )\beta \bar{I}\\ & \;+(\mu +\eta \bar{R})\left((\mu +p)+\eta \bar{R}\right)\left((\mu +p)+\beta \bar{I}\right)]+[(\mu +p)(\alpha +\mu +\gamma )\\ & \;+(\mu +\eta \bar{R})\left((\mu +\eta )+\beta \bar{I}\right)+(\epsilon +\mu +\sigma \beta )\eta \bar{R}]x_{11}\\ & =\left|\begin{array}{ccc} x_{1}-x_{5} & x_{3}-x_{7} & 0\\ 1 & x_{2}-x_{6} & x_{4}-x_{8}\\ 0 & x_{1}-x_{5} & x_{3}-x_{7} \end{array}\right|\\ & =(x_{1}-x_{5})[(x_{2}-x_{6})(x_{3}-x_{7})-(x_{1}-x_{5})(x_{4}-x_{8})]{\left(x_{3}-x_{7}\right)}^{2}. \end{array} \end{array}$$

Let $m=\mu +\eta \bar{R}$, $n=(\mu + p)+\beta \bar{I}$. Thus,

$$\begin{aligned} x_{11} =&\bigl[(\alpha + \mu + \gamma ) m\bigl(n-(\mu + p)\bigr)+\eta ( \epsilon +\mu + \sigma \beta )\bar{R}n+\eta (\epsilon + \mu + \sigma \beta )\bar{R}(\alpha + \mu + \gamma )\bigr] \\ &{}\bigl((\alpha + \mu + \gamma ) n^{2}+m n^{2}+(\alpha + \mu + \gamma )^{2}n+(\alpha + \mu + \gamma ) mn \\ &{}+(\mu + p) (\alpha + \mu + \gamma ) m+m^{2}n+ ( \epsilon +\mu + \sigma \beta ) m \eta \bar{R}\bigr) \\ &{}- (\epsilon +\mu + \sigma \beta ) (\mu + p) (\alpha + \mu + \gamma ) \eta \bar{R}\bigl(n^{2}+(\alpha + \mu + \gamma )^{2}+2( \alpha + \mu + \gamma )n \\ &{}+m^{2}+2mn+2m(\alpha + \mu + \gamma )\bigr) \\ =&(\alpha + \mu + \gamma )m\bigl(n-(\mu + p)\bigr)\bigl((\alpha + \mu + \gamma ) n^{2}+mn^{2}+( \alpha + \mu + \gamma )^{2}n \\ &{}+(\alpha + \mu + \gamma ) mn+(\mu + p) ( \alpha + \mu + \gamma ) m+m^{2}n+ (\epsilon +\mu + \sigma \beta ) m \eta \bar{R}\bigr) \\ &{}+ (\epsilon +\mu + \sigma \beta ) \eta \bar{R} (\alpha +\mu + \gamma )^{2}m\bigl(n-(\mu + p)\bigr) \\ &{}+ ( \epsilon +\mu + \sigma \beta ) (\alpha + \mu + \gamma )^{3} \eta \bar{R}(n-d) \\ &{}+2 (\epsilon +\mu + \sigma \beta ) \eta \bar{R} (\alpha + \mu + \gamma )^{2}n\bigl(n-(\mu + p)\bigr) \\ &{}+ ( \epsilon +\mu + \sigma \beta ) \eta \bar{R} (\alpha + \mu + \gamma ) m^{2}\bigl(n-(\mu + p)\bigr) \\ &{}+2 (\epsilon +\mu + \sigma \beta ) \eta \bar{R} (\alpha + \mu + \gamma ) mn\bigl(n-(\mu + p)\bigr) \\ &{}+ ( \epsilon +\mu + \sigma \beta ) \eta \bar{R} (\alpha + \mu + \gamma )^{2}m\bigl(n-(\mu + p)\bigr) \\ &{}+ (\epsilon +\mu + \sigma \beta ) \eta \bar{R}mn^{3}+ (\epsilon +\mu + \sigma \beta ) \eta \bar{R}(\mu + p) (\alpha + \mu + \gamma ) mn \\ &{}+ ( \epsilon +\mu + \sigma \beta ) \eta \bar{R}m^{2}n^{2}+ ( \epsilon +\mu + \sigma \beta )^{2}m \eta ^{2}\bar{R}^{2}n \\ &{}+ (\epsilon +\mu + \sigma \beta )^{2} \eta ^{2}\bar{R}^{2} ( \alpha + \mu + \gamma )m. \end{aligned}$$

We have $x_{11}>0$, since $n-(\mu + p)>0$.

$$x_{12}=\left|\begin{array}{cccc} x_{1}-x_{5} & x_{3}-x_{7} & 0 & 0\\ 1 & x_{2}-x_{6} & x_{4}-x_{8} & 0\\ 0 & x_{1}-x_{5} & x_{3}-x_{7} & 0\\ 0 & 1 & x_{2}-x_{6} & x_{4}-x_{8} \end{array}\right|=a_{4}x_{11}.$$

Taking note of $a_{4}= (\epsilon +\mu + \sigma \beta ) (\mu + p) ( \alpha + \mu + \gamma ) \eta \bar{x}$, it is anything but difficult to acquire that $x_{12}>0$. Subsequently, the real parts are negative in (14). This completes the verification of Theorem 3.2. Here the roots of $\Omega (\lambda )=0$ have negative real roots. Hence, there exists a $\tau _{0}>0$ to such that $\tau \in [0,\tau _{0})$ in (13), and we have

15 $$\begin{aligned} \Omega (\lambda )=0,\qquad \operatorname{Re}( \lambda )< 0 \quad \text{for } \tau \in [0,\tau _{0}), \end{aligned}$$

moreover, when $\tau =\tau _{0}$, $\operatorname{Re}(\lambda )<0$. To decide on this $\tau _{0}$ and the related simply $\phi _{0}i(\phi _{0}>0)$ imaginary roots, we understand (13) with $\lambda =\phi _{0}i$. For straightforwardness, we use $\tau, \phi $ rather than $\tau _{0}, \phi _{0}$. From (13), we have

16 $$\begin{aligned} &\phi ^{4}-x_{1} \phi ^{3}i-x_{2}\phi ^{2}+x_{3}\phi i+x_{4}-\bigl(-x_{5} \phi ^{3}i-x_{6} \phi ^{2}+x_{7} \phi +x_{8}\bigr) \\ &\quad{}(\cos \phi \tau -i\sin \phi \tau )=0. \end{aligned}$$

Comparing the coefficients of real and imaginary parts, we get

17 $$\begin{aligned} &\textstyle\begin{cases} (x_{8}-x_{6} \phi ^{2})\cos \phi \tau +(x_{7} \phi -x_{5} \phi ^{3})\sin \phi \tau =\phi ^{4}-x_{2} \phi ^{2}+ x_{4}, \\ (x_{5} \phi ^{3}-x_{7} \phi )\cos \phi \tau +(x_{8}-x_{6} \phi ^{2})\sin \phi \tau =x_{1} \phi ^{3}-x_{3} \phi, \end{cases}\displaystyle \\ &\cos \phi \tau =\frac{1}{\Delta } \begin{vmatrix} \phi ^{4}-x_{2} \phi ^{2}+x_{4} & x_{7} \phi -x_{5} \phi ^{3} \\ x_{1} \phi ^{3}-x_{3} \phi & x_{8}-x_{6} \phi ^{2} \end{vmatrix} \\ &\phantom{\cos \phi \tau}=\frac{1}{\Delta } \bigl[(x_{1} x_{5}-x_{6}) \phi ^{6}+(x_{8}+x_{2} x_{6}-x_{1} x_{7}-x_{3} x_{5}) \phi ^{4} \\ &\phantom{\cos \phi \tau=}{}+(x_{3} x_{7}-x_{2} x_{8}-x_{4} x_{6})\phi ^{2}+x_{4} x_{8}\bigr] \\ &\phantom{\cos \phi \tau}=\frac{1}{\Delta } \bigl(c_{1} \phi ^{6}+c_{2} \phi _{4}+c_{3} \phi ^{2}+c_{4} \bigr), \\ &\sin \phi \tau =\frac{1}{\Delta } \begin{vmatrix} x_{8}-x_{6} \phi ^{2} & \phi ^{4}-x_{2} \phi ^{2}+x_{4} \\ x_{5} \phi ^{3}-x_{7} \phi & x_{1} \phi ^{3}-x_{3} \phi \end{vmatrix} \\ &\phantom{\sin \phi \tau }= \biggl(-\frac{\phi }{\Delta } \biggr) \bigl(x_{5} \phi ^{6} + (x_{1} x_{6}-x_{7}-x_{2} x_{5}) \phi ^{4} + (x_{2} x_{7}+ x_{4} x_{5}-x_{3} x_{6}-x_{1} x_{8}) \phi ^{2} \\ &\phantom{\sin \phi \tau =}{}+ (x_{3} x_{8}-x_{4} x_{7})\bigr) \\ &\phantom{\sin \phi \tau }=-\frac{\phi }{\Delta }\bigl(x_{9} \phi ^{6}+ x_{10} \phi ^{4}+ x_{11} \phi ^{2}+ x_{12}\bigr), \end{aligned}$$

where

$$\begin{aligned} \Delta ={}&\begin{vmatrix} x_{8}-x_{6} \phi ^{2} & x_{7} \phi -x_{5} \phi ^{3} \\ x_{4} \phi ^{3}-x_{7} \phi & x_{8}-x_{6} \phi ^{2} \end{vmatrix} \\ ={}&\bigl(x_{8}-x_{6} \phi ^{2} \bigr)^{2}+\bigl(x_{7}-x_{5} \phi ^{3} \bigr)^{2}=x_{5} \phi ^{6}+(x_{6}-2x_{5} x_{7})\phi ^{4}+\bigl(x_{7}^{2}-2x_{6} x_{8}\bigr)\phi ^{2} \\ &{}+x_{8}^{2}=\bigl(e_{1} \phi ^{6}+e_{2} \phi ^{4}+e_{3} \phi ^{2}+e_{4}\bigr)>0. \end{aligned}$$

Here $\sin ^{2}\phi \tau +\cos ^{2}\phi \tau =1$, it follows that

18 $$\begin{aligned} \phi ^{14}+x_{13} \phi ^{12}+x_{14} \phi ^{10}+x_{15} \phi ^{8}+x_{16} \phi ^{6}+x_{17} \phi ^{4}+x_{18} \phi ^{2}+x_{19}=0, \end{aligned}$$

where

$$\begin{aligned}& x_{13}=\frac{1}{x_{9}^{2}}\bigl(c_{1}^{2}+2x_{9} x_{10}-e_{1}^{2}\bigr), \\& x_{14}=\frac{1}{x_{9}^{2}}\bigl(2c_{1}c_{2}+x_{10}^{2}+2x_{9} x_{11}-2e_{1}e_{3}\bigr), \\& x_{15}=\frac{1}{x_{9}^{2}}\bigl(c_{2}^{2}+2c_{1}c_{3}+2x_{9} x_{12}+2x_{10} x_{11}-e_{2}^{2}-2e_{1}e_{3}\bigr), \\& x_{16}=\frac{1}{x_{9}^{2}}\bigl(2c_{1}c_{4}+2c_{2}c_{3}+x_{11}^{2}+2x_{10} x_{12}-2e_{1}e_{4}-2e_{2}e_{3}\bigr), \\& x_{17}=\frac{1}{x_{9}^{2}}\bigl(c_{3}^{2}+2c_{2}c_{4}+2 x_{11} x_{12}-e_{3}^{2}-2e_{2}e_{4}\bigr), \\& x_{18}=\frac{1}{x_{9}^{2}}\bigl(2c_{3}c_{4}+x_{12}^{2}-2e_{3}e_{4}\bigr), \\& x_{19}=\frac{1}{x_{9}^{2}}\bigl(c_{4}^{2}-e_{4}^{2}\bigr). \end{aligned}$$

Denoting: $x=\phi ^{2}$, (18) becomes

19 $$\begin{aligned} x^{7}+x_{13} x^{6}+x_{14} x^{5}+x_{15} x^{4}+x_{16} x^{3}+x_{17} x^{2}+x_{18} x+x_{19}=0. \end{aligned}$$

First, $x=0$ is not a root of (19) if $x_{8} \neq 0$. There is no positive real root in (19). Therefore $\phi =\sqrt{x}$ does not get the solution. Hence the bifurcation parameter *τ* does not occur and a Hopf bifurcation is not evaluated. Equation (19) always has positive real roots. Let the hypothesis be as follows:

$(\Omega _{1})$:
Equation (19) has possibly one positive real root;
$(\Omega _{2})$:
$\Lambda \stackrel{\Delta }{=}[4\phi ^{6}+3(x_{1}^{2}-2x_{2}-x_{5}^{2}) \phi ^{4}+2(x_{2}^{2}-x_{6}^{2}+2x_{4}+x_{5} x_{7}-2x_{1} x_{3})\phi ^{2}+x_{3}^{2}-x_{7}^{2}+2x_{6} x_{8}-2x_{2} x_{4}]>0$ for any $\phi >0$.

Let $x_{0}$ be the positive roots of (19), denoting $\phi _{0}=\sqrt{x_{0}}$. From the above, we get

$$ \tau _{j}=\frac{1}{\phi _{0}} \biggl(\cos ^{-1} \biggl( \frac{c_{1} \phi _{0}^{6}+c_{2} \phi _{0}^{4}+c_{3} \phi _{0}^{2}+c_{4}}{e_{1} \phi _{0}^{6}+e_{2} \phi _{0}^{4}+e_{3} \phi _{0}^{2}+e_{4}} \biggr)+2j\pi \biggr),\quad j=0,1,2,3,\ldots, $$

and

$$ \tau _{0}=\frac{1}{\phi _{0}} \cos ^{-1} \biggl( \frac{c_{1} \phi _{0}^{6}+c_{2} \phi _{0}^{4}+c_{3} \phi _{0}^{2}+c_{4}}{e_{1} \phi _{0}^{6}+e_{2}\phi _{0}^{4}+e_{3} \phi _{0}^{2}+e_{4}} \biggr),\quad j=0. $$

The set of ordered pair is $(\phi _{0},\tau _{0})$ to find the polynomial roots of (13) in a neighborhood of $\tau _{0}$ and differentiating with respect to *τ*, we get

20 $$\begin{aligned} \biggl[\frac{d\lambda }{d\tau } \biggr]^{-1}= \frac{-(4\lambda ^{3}+3x_{1}\lambda ^{2}+2x_{2}\lambda +x_{3})e^{\lambda }\tau }{\lambda (x_{5}\lambda ^{3}+x_{6}\lambda ^{2}+x_{7}\lambda +x_{8})}+ \frac{3x_{5}\lambda ^{2}+2x_{6}\lambda +x_{7}}{\lambda (x_{5}\lambda ^{3}+x_{7}\lambda ^{2}+x_{7}\lambda +x_{8})}- \frac{\tau }{\lambda }. \end{aligned}$$

Letting (20), we have

$$\begin{aligned} \operatorname{Re} \biggl[\frac{d\lambda }{d\tau } \biggr]^{-1}={}& \frac{1}{\phi \nabla } \bigl\{ \bigl(3x_{1} \phi ^{2}-x_{3} \bigr)\bigl[\bigl(x_{5} \phi ^{3}-x_{7}\phi \bigr)\cos \phi \tau +\bigl(x_{8}-x_{8} \phi ^{2} \bigr)\sin \phi \tau \bigr] \\ &{}+\bigl(4\phi ^{3}-2x_{2} \phi \bigr)\bigl[ \bigl(x_{8}-x_{6} \phi ^{2}\bigr)\cos \phi \tau - \bigl(x_{5} \phi ^{3}-x_{7} \phi \bigr)\sin \phi \tau \bigr] \\ &{}+\bigl(x_{7}-3x_{5} \phi ^{2}\bigr) \bigl(x_{5} \phi ^{3}-x_{7} \phi \bigr)+2x_{6} \phi \bigl(x_{8}-x_{6} \phi ^{2}\bigr)\bigr\} \\ ={}&\frac{1}{\nabla }\bigl[4\phi ^{6}+3\bigl(x_{2}^{2}-2x_{2}-x_{5}^{2} \bigr)\phi ^{4}+2\bigl(x_{2}^{2}-x_{6}^{2}+2x_{4}+2x_{5} x_{7}-2x_{1} x_{3}\bigr)\phi ^{2} \\ &{}+x_{3}^{2}-x_{7}^{2}+2x_{6} x_{8}-2x_{2} x_{4}\bigr], \end{aligned}$$

where $\nabla =(x_{5} \phi ^{3}-x_{7} \phi )^{2}+(x_{8}-x_{6} \phi ^{2})^{2}>0$. If $(\Omega _{2})$ is satisfied, then $\text{(20)}>0$ will hold for any $\phi >0$.

So, $\operatorname{sign} \{ \operatorname{Re} [\frac{d\lambda }{d\tau } ] |\tau = \tau _{0} \} =\operatorname{sign} \{ \operatorname{Re} [\frac{d\lambda }{d\tau } ] |\tau =\tau _{0} \} \stackrel{\Delta }{=}\operatorname{sign} (\cdot)=1$.

Therefore, the roots of (14) have negative real parts. If $\tau =\tau _{0}$, then other negative real roots have $\Omega (\lambda )=0$. In (14), if $\tau \in [0,\tau _{0})$ and $(\Omega _{1}, \Omega _{2})$ assumption, then the COVID-19 infection is stable. Similarly, if $\tau >\tau _{0}$, then the COVID-19 infection is unstable and Eq. (2) shows bifurcation at $\tau =\tau _{0}$. □

## Analysis of sensitivity parameters

The effects of changing parameter values on the functional value of the reproduction number $R_{0}$ are obtainable in this section. The essential parameter must be found, which could be an important threshold for disease management. The algebraic representations of the sensitivity index of $R_{0}$ to the parameters $\eta, \beta,\sigma, \gamma, \alpha, b, p$ are as follows:

$$\begin{aligned} &\frac{\partial R_{0}}{\partial \beta } = \frac{2\beta \sigma b}{\mu (\mu +p)(\mu +\gamma +\alpha )}, \qquad\frac{\partial R_{0}}{\partial b} = \frac{\beta (\eta +\sigma \beta )}{\mu (\mu +p)(\mu +\gamma +\alpha )}, \\ &\frac{\partial R_{0}}{\partial \eta } = \frac{\beta b}{\mu (\mu +p)(\mu +\gamma +\alpha )},\qquad \frac{\partial R_{0}}{\partial p} = \frac{-\beta b(\eta +\sigma \beta )}{\mu (\mu +p)^{2}(\mu +\gamma +\alpha )}, \\ &\frac{\partial R_{0}}{\partial \sigma } = \frac{\beta ^{2} \sigma }{\mu (\mu +p)(\mu +\gamma +\alpha )},\qquad \frac{\partial R_{0}}{\partial \alpha } = \frac{-\beta b(\eta +\sigma \beta )}{\mu (\mu +p)(\mu +\gamma +\alpha )^{2}}, \\ &\frac{\partial R_{0}}{\partial \gamma } = \frac{-\beta b(\eta +\sigma \beta )}{\mu (\mu +p)(\mu +\gamma +\alpha )^{2}}. \end{aligned}$$

It is concluded that some partial derivatives are positive, with the increase of any of the above positive value parameters $\eta, \beta, \sigma $ the basic reproductive number $R_{0}$ increases. The elasticity is estimated with the proportional response to the proportional perturbation. We have

$$\begin{aligned} &E_{\beta }=\frac{\beta }{R_{0}}\frac{\partial R_{0}}{\partial \beta }= \frac{2\beta \sigma }{(\eta +\sigma \beta )}=0.9804,\qquad E_{\eta }= \frac{\eta }{R_{0}}\frac{\partial R_{0}}{\partial \eta }= \frac{\eta }{(\eta +\sigma \beta )}=0.5098, \\ &E_{\sigma }=\frac{\sigma }{R_{0}} \frac{\partial R_{0}}{\partial \sigma }= \frac{\beta \sigma }{(\eta +\sigma \beta )}=0.4901. \end{aligned}$$

From the above expressions, it is observed that $E_{\eta }, E_{\beta }$ and $E_{\sigma }$ are positive. This implies an increase in the parameters $\eta, \beta $ and *σ* leads to an increase in the value of the basic reproduction number $R_{0}$. The smallest change in these parameters can cause a high variation in the basic reproduction number. A very sensitive parameter should be carefully calculated since a slight variation can lead to major quantitative changes in the system.

## Numerical experiment

**Figure 1:**
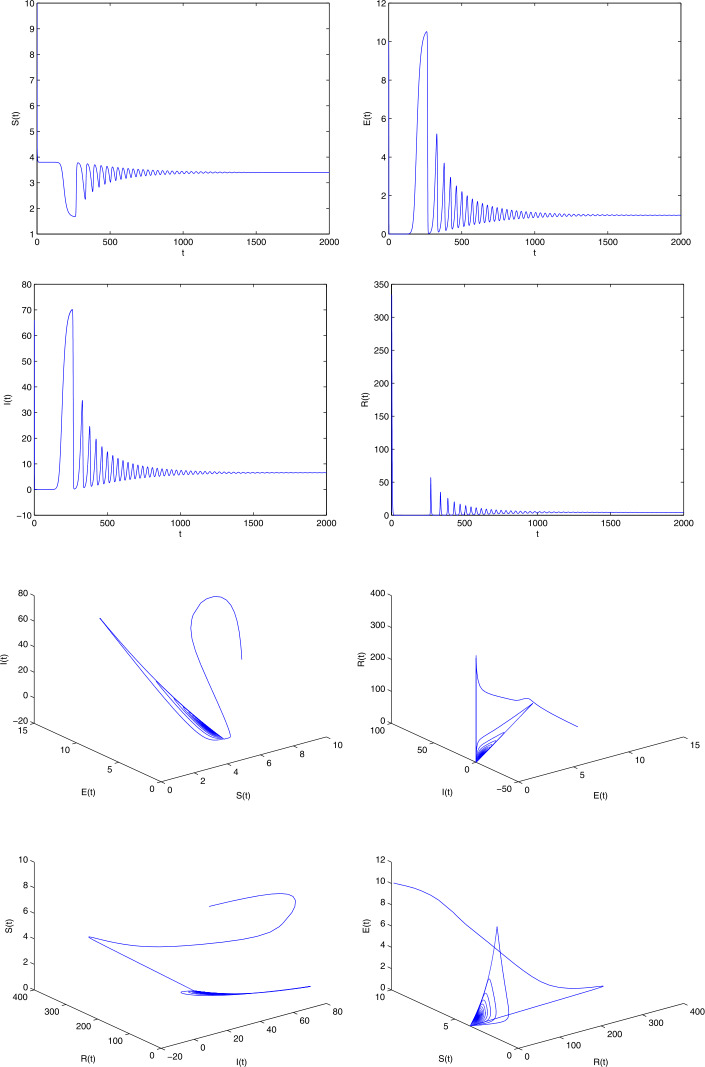
The phase diagrams of the system (2) is asymptotically stable when $\tau = 0.9$

**Figure 2:**
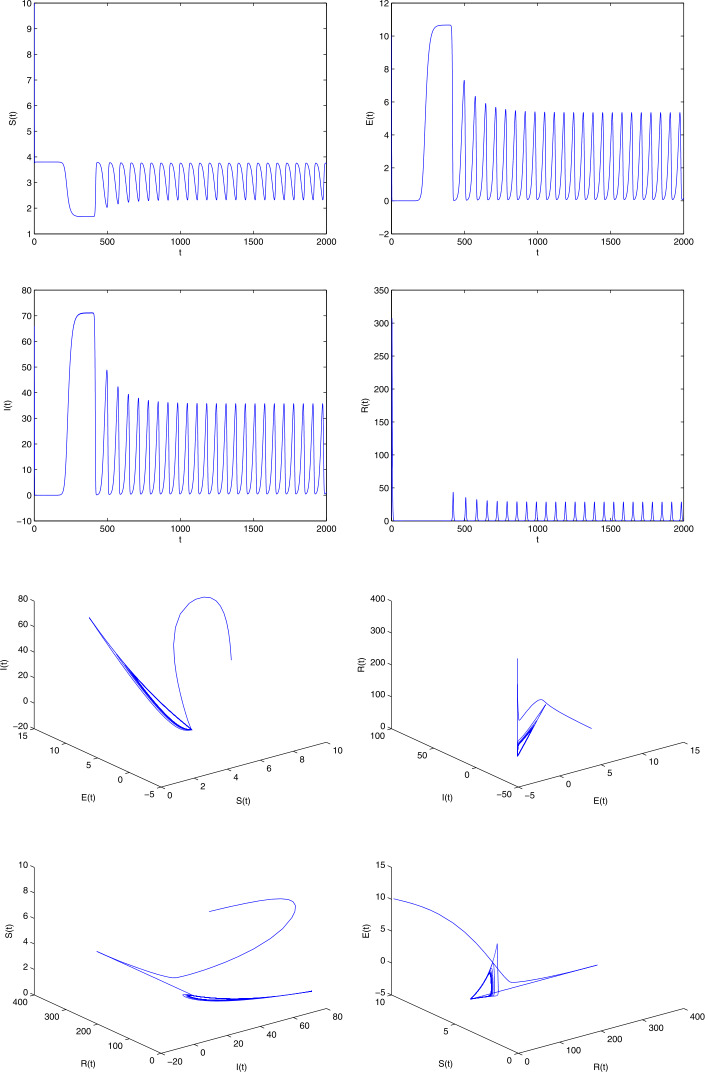
The phase diagrams of the system (2) undergoes Hopf bifurcation when $\tau = 2$

**Figure 3:**
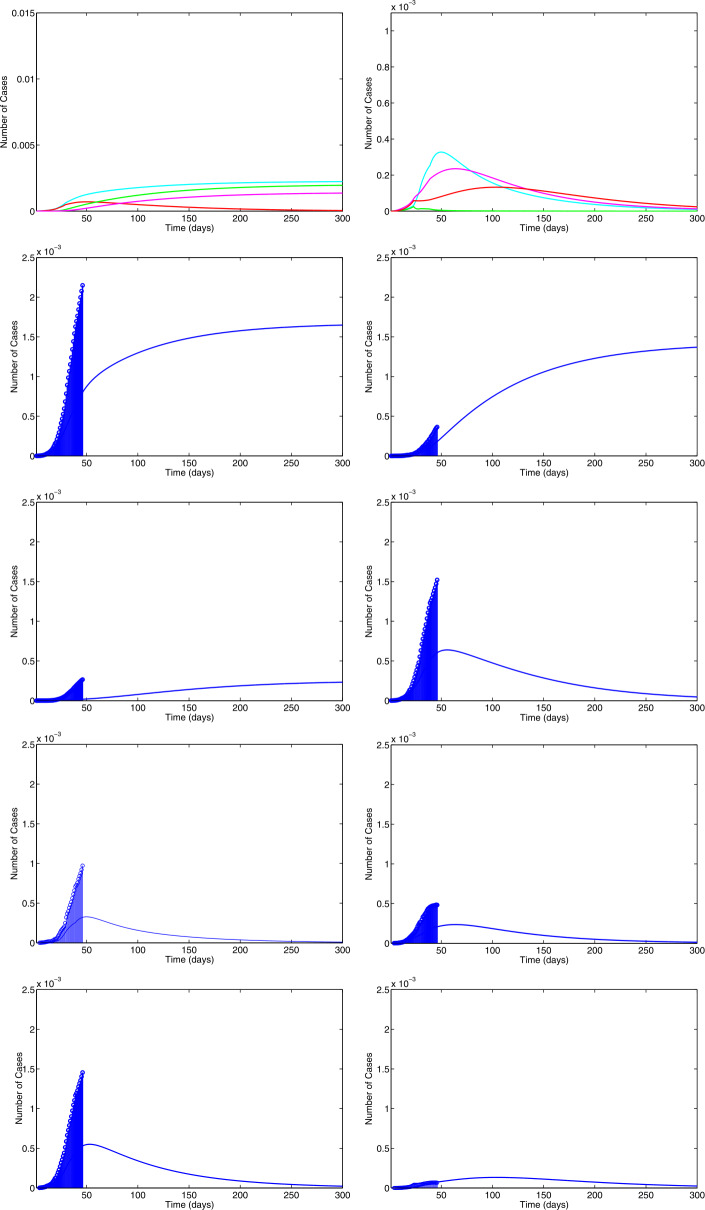
The epidemic numerical simulations of (2) from real-life data

**Figure 4:**
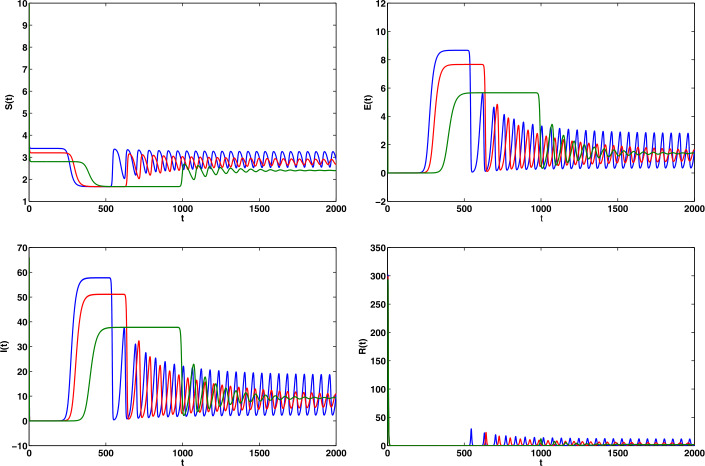
The phase diagram of S, E,I, R for different transmission rates at $\tau = 2$, the remaining parameters are taken $(\beta =0.05, 0.10 \& 0.15)$ as indicated as above

The COVID 19 model has unspecified parameters of SEIR model. The model’s identities are investigated by the iterative algorithm and the parameter values (Age-standardized SEIR model) of the real-life data for the COVID 19 pandemic in India should be determined from these model values. COVID 19 data are therefore important in developing and validating the nonlinear ODE. Let us consider the parameters $b = 0.5, \gamma = 0.008, \epsilon = 0.1, \mu = 0.0018, p = 0.5, \beta = 0.1923, \eta = 0.1, \sigma = 0.5, \alpha =0.5$ with $(S(0), E(0), I(0), R(0))$ [34]. In the event that (19) has no positive roots, at that point the COVID-19 infection present equilibrium is locally asymptotic stable. On the off chance that $R_{0} = 2.47$, at that point the COVID-19 disease presents equilibrium $E = (3.1, 1.4, 10.01, 2.01)$. From (19), we have

$$ x^{7} + 500.01 x^{6} + 10\text{,}025 x^{5} + 23 \text{,}423 x^{4} + 4099 x^{3} + 28.1 x^{2} + 0.1 x + 6.110^{-5} = 0, $$

having real negative roots. Therefore the equilibrium is locally asymptotic stable and it represents a Hopf bifurcation. Obviously, $R_{0} = 1.92$, and the COVID-19 infection presenting an equilibrium has $E = (3.01, 1.3, 8.1, 3.3)$. From (19), we have

$$ x^{7} + 477.2304 x^{6} + 47\text{,}123 x^{5} + 33\text{,}257 x^{4} + 5008 x^{3} + 27.1 x^{2} - 1.3 x - 0.001 =0, $$

having positive real roots and others having negative real roots. Accordingly, $\phi _{0} = \sqrt{x}= 0.1$ It is not hard to evaluate the bifurcation stationary value to be $\tau _{0} = 1.96$. Also, it is anything but difficult to prove that $\Lambda = 2.8 > 0$, i.e., $(\Omega _{2})$ is fulfilled. The phase diagram of the system (2) is asymptotic stable when $\tau = 0.9 < \tau _{0}$ (see Fig. 1). Also, the phase diagrams of the system (2) undergoes a Hopf bifurcation when $\tau = 2 > \tau _{0}$ (see Fig. 2). We utilize the serious cases and deaths in the individual response work, rather than deaths as it were. We additionally increment the power of the legislative activity to such an extent that the model results to a great extent in a coordinate of the watched, with a revealing proportion. In Fig. 3 shows the numerical simulations and calculated the different $R_{0}$ values such as 2.0317, 1.2922, 1.4809, 1.5972 and 0.9844 from the real-life data already published (WHO). The range of the $R_{0}$ values lies between 0.9844 and 2.0317. The time plots of SEIR COVID-19 model for different recruitment rate at $\tau = 2$ (see Fig. 4). To be specific just the extent of the model’s created cases will be accounted for as a general rule. Consequently it would concern testing given a generally brief time frame arrangement, and a few other obscure parameters are to be assessed. Figure 4 depicts how decreasing the transmission rate can change the system dynamics from the limit cycle to stable focus. It implies that without the Hopf bifurcation, the system is stable and controllable. Based on the sensitivity analysis value, decreasing the transmission rate can change the dynamics of the system from a limit cycle to a stable focus.

## Conclusion

There is a shortage of epidemiological information about the rising coronavirus, which would be of essential significance to structure and executing auspicious, specially appointed viable general well being intercessions, isolation and travel limitations. We have contemplated a general SEIR model of COVID-19 infection with delay. If $R_{0} < 1$, then stability of the disease-free equilibrium is derived by Lyapunov techniques. Furthermore as regards the effects of time delay $\tau = 0$, the COVID-19 infection is either absent or presents an equilibrium when $R_{0} > 1$. Here $1 < R_{0} < 1 + \frac{\beta ^{2} (\eta + \sigma \beta )^{2} (\epsilon +\mu + \sigma \beta )}{\mu p (\mu + p) (\alpha + \mu + \gamma )^{2}}$, then $E_{1}$ is stable. If $R_{0} > 1 + \frac{\beta ^{2} (\eta + \sigma \beta )^{2} (\epsilon +\mu + \sigma \beta )}{\mu p (\mu + p) (\alpha + \mu + \gamma )^{2}} $, then $E_{1}$ is unstable. Hence, $\tau > 0$, $1 < R_{0} < 1 + \frac{\beta ^{2} (\eta + \sigma \beta )^{2} (\epsilon +\mu + \sigma \beta )}{\mu p (\mu + p) (\alpha + \mu + \gamma )^{2}} $, $E_{1}$ is stable. The basic reproductive ratio $R_{0} > 1 + \frac{\beta ^{2} (\eta + \sigma \beta )^{2} (\epsilon +\mu + \sigma \beta )}{\mu p (\mu + p) (\alpha + \mu + \gamma )^{2}} $, if the susceptible cells birth rate is high. Therefore the linearized system of (2) has no real positive roots and we have stability. The polynomial equation (19) has a single real positive root when $\tau < \tau _{0}$. The COVID-19 infection presenting equilibrium is stable. If $\tau > \tau _{0}$, the equilibrium solutions are unstable and a Hopf bifurcation occurs. Supposing Eq. (19) has more than one positive root, it does not exit. In the future, further investigation is needed to this system. The controlling of the reproduction number ratios proposes that the outbreak might be more genuine than what has been accounted for up until now, given the specific period of expanding social contacts, justifying powerful, severe general well being measures planned to relieve the weight produced by the spreading of the new infection. Finally, as regards the transmission rate it can be concluded that the system dynamics can be modified by decreasing its value from a limit cycle to a stable focus. Important measures to reduce the proportion of people susceptible to infection can be taken through increasing their immunity, quarantining infectious people, and decreasing their interaction with susceptible people. By using the least square approach, we used the descendant gradient model and identified the nearly approximate value of the parameters. The most important factor in preventing the spread of the virus locally is to empower the citizens with the right information and taking precautions as per the advisories being issued by the Ministry of Health & Family Welfare.
